# Combined omics data reveal multilevel response to water deficit in grapevine disease-resistant varieties

**DOI:** 10.3389/fpls.2026.1818883

**Published:** 2026-07-08

**Authors:** Rebeka Strah, Rachele Falchi, Chao Song, Andrej Blejec, Živa Ramšak, Kristina Gruden, Enrico Peterlunger, Aaron Fait, Maruša Pompe-Novak, Paolo Sivilotti

**Affiliations:** 1 National Institute of Biology, Department of Biotechnology and Systems Biology, Ljubljana, Slovenia; 2 Jožef Stefan International Postgraduate School, Ljubljana, Slovenia; 3 Department of Agricultural, Food, Environmental and Animal Sciences, University of Udine, Udine, Italy; 4 Albert Katz International School for Desert Studies, Jacob Blaustein Institutes for Desert Research, Ben-Gurion University of the Negev, Beersheba, Israel; 5 Department of Biology, Biotechnical Faculty, University of Ljubljana, Ljubljana, Slovenia; 6 Faculty for Viticulture and Enology, University of Nova Gorica, Vipava, Slovenia

**Keywords:** caffeoylquinic acid, disease-resistant variety, drought tolerance, grapevine, multiomics integration, water deficit

## Abstract

Climate change is intensifying drought stress in viticultural regions, threatening grapevine productivity and quality. New genotypes previously bred for disease resistance are an untested resource regarding their water deficit tolerance. Field experiments were conducted over two seasons, applying well-watered and water-stress treatments in two disease-resistant varieties, Fleurtai and Cabernet Volos. Physiological measurements, RNA sequencing, and GC-MS-based metabolite profiling of leaves were integrated, correlating gene expression and metabolite accumulation with stem water potential (Ψ_S_) using a factorial analyses design by FaDSeqSes script. We identified three categories of response: (1) genes and metabolites similarly regulated by water deficit in both genotypes, belonging to several different pathways (such as carbohydrate metabolism, amino acid metabolism, secondary metabolism, and hormone metabolism), among which sugar metabolism was one of the most striking ones, including changes in accumulation of raffinose and galactinol, and induction of genes coding for their synthesis; (2) responses specific to Cabernet Volos, characterized by downregulation of kinase and receptor genes likely to be involved in shutting down biotic defense response; and (3) responses specific to Fleurtai, including accumulation of caffeoylquinate and upregulation of genes involved terpene synthesis and in ABA regulation. We found that each genotype has an individual way to combat water deficits; Cabernet Volos accumulates more osmoprotectant compounds at a constant and higher level, while Fleurtai synthesizes these compounds as needed when stress occurs. This study underscores overall grapevine responses to water deficits, as well as the contribution of the genotype.

## 1 Introduction

Climate change leads to increasing temperatures, shifting precipitation patterns, and an intensification in the frequency of extreme weather events. In particular, persistent drought is threatening many ecosystems (Intergovernmental Panel on Climate Change (IPCC), 2022).

Viticulture is drastically affected by climate change and many viticultural areas already suffering from seasonal drought and water availability could be further affected by the worsening situation (Sgubin et al., 2023). The Mediterranean, a hotspot for wine production, is one of the world’s regions most exposed to these risks (Prada et al., 2024).

Prolonged water deficit, when midday stem water potential (Ψ_s_) drops below -0.8 MPa, can negatively affect grape yields and quality (Chaves et al, 2010; Hochberg et al., 2013; Savoi et al., 2016) and have persistent effects on grapevine water use (Hochberg et al., 2023). Although patterns of stress may vary widely between environments, it is still debated to what extent variation in water-use regulation among grapevine varieties results from innate genotypic differences, environmental factors or a combination of both (Hochberg et al., 2015).

Changes in local climate call for use of water deficit resistant varieties (Pedneault and Provost, 2016). However, many recently developed grapevine cultivars are the result of long breeding programs that were focused on disease resistance. The most common fungal pathogens have been a particular focus, with the aim to reduce the use of pesticides in viticulture (Bove et al., 2019; Sargolzaei et al., 2020). Several disease-resistant varieties with high grape quality were produced by interspecific cross-breeding between *V. vinifera* and *Vitis* spp., that carry high resistance to fungal diseases (Bove et al., 2019; Coleman et al., 2009; Di Gaspero et al., 2012; Venuti et al., 2013). These already existing varieties must be tested for their response to water deficit conditions. Recently, berry primary metabolites and thiol precursors have been investigated in six disease-resistant varieties under different water regimes in field conditions (de Almeida et al., 2024). Water deficit significantly decreased the accumulation of all measured metabolites, regardless of genotype. Disease-resistant varieties remain a largely unexplored pool of drought resistant traits.

High-throughput approaches have greatly aided our understanding of how plants interact with their environment. The plants rely on complex regulatory networks to cope with biotic and abiotic stresses. Overlapping responses to these stress types involve the production of ROS, activation of hormones, reorganization of the metabolic network, changes in expression of specific transcription factor families, expression of specific small RNAs, and signaling cascades involving kinases (Leisner, 2023). Interactions between biotic and abiotic stress in grapevine have also been studied, but the observations vary from studies that show increased sensitivity of infected plants due to stomatal deregulation (Allègre et al., 2007), to increased resistance to *Plasmopara viticola* in drought conditions due to altered ABA signaling (Heyman et al., 2021). These large discrepancies can occur because of the different studied cultivars as well as experimental setups. Several high-throughput analyses can be combined to further elucidate how different grapevine cultivars fare in the changing environment. The current study aims to gain insight into the response of two disease-resistant varieties, Cabernet Volos and Fleurtai, to the occurrence of water stress. These varieties originate from crosses with a cultivar of international interest (Cabernet Sauvignon), producing the red Cabernet Volos, and crosses with a minor local variety with high oenological potential in northeastern Italy (Tocai Friulano), producing Fleurtai (Bavaresco et al., 2015). We implemented metabolite profiling combined with transcriptomic analysis to investigate the behavior of these genotypes during water stress. The information obtained could lead to optimized irrigation management for these grape varieties, providing a tangible boost to profitability and sustainability in viticulture.

## 2 Materials and methods

### 2.1 Plant material and experimental design

The study was carried out in 2018 and 2019 seasons at the experimental farm “A. Servadei” of the University of Udine, in a vineyard planted in 2011 with two grapevine interspecific hybrids: the white berry genotype Fleurtai (Tocai friulano x 20/3) and the red berry genotype Cabernet Volos (Cabernet Sauvignon x 20/3). Both varieties were selected in a breeding program for the introgression of resistant genes to powdery and downy mildew and were registered for cultivation in 2015 in Italy (Bavaresco et al., 2015). The vines were arranged in two rows North-South oriented and pruned to a single Guyot training system with a vertical shoot positioned (VSP) trellis system retaining 8–9 buds/vine. For each genotype, 24 plants were considered and divided into two plots, assigned to the well-watered (WW) and to the water-stress (WS) treatment, respectively. Within each treatment, four plots with three vines in each were considered in both years of experiment for both physiological measurements and sampling. Irrigation of WW treatment was applied every week to maintain stem water potential (Ψ_s_) values above -0.6 MPa for the whole season, while WS was obtained by withholding irrigation starting at 10 days after flowering; emergency irrigation was applied to WS plants only when Ψ_s_ reached -1.2 MPa for Fleurtai and -1.4 MPa for Cabernet Volos. The ground was covered with ethylene-vinyl-acetate film to ensure proper water deficit in case of summer rain. The film was further covered by 3–4 cm of soil to avoid microclimate alteration due to soil heating and radiation reflection.

In both seasons, measurements of plant water status and gas exchanges were performed around midday in ten time points evenly distributed during the periods of water stress. Water potential (Ψ_s_) was determined according to the procedure described in Braidotti et al. (2024). Moreover, stomatal conductance (g_S_) and phososynthesis (A_n_) were measured in sun-exposed and fully developed leaves (one leaf per plot, four leaves per treatment) with a portable photosynthesis system LI-6400 XT (LiCor, Inc., NE, 119 USA), using a constant light intensity (1000 μmol m-^2^s^-1^), CO_2_ concentration (400 μmol mol^-1^), and 120 ambient humidity. Moreover, water use efficiency (WUE_i_) was computed by rating g_S_ and A_n_.

During both years, three leaves per plot were collected by selecting fully expanded leaves between the 6th and 10th nodes, wrapped in aluminum foil, and flash-frozen in liquid nitrogen and stored at -80 °C. Thereafter, leaf samples were ground to a fine powder and stored at -80 °C for subsequent analysis. Samples were collected at six time points in both seasons from the beginning of water stress imposition (T_0_) to harvest, namely 12, 18, 31, 50, 58, and 65 days after T_0_ in 2018, and 16, 28, 37, 50, 57, and 63 days after T_0_ in 2019. All samples were processed and analyzed using a GC-MS based metabolite profiling protocol (Hochberg et al., 2013), while samples from only five time points (three in 2018 and two in 2019) were selected for transcriptomic analysis based on Ψ_s_ values. In 2018, samples from 10, 34, and 67 days after the beginning of water deficit were selected. In the following season, the temperatures in June and July were much higher than in 2018, thus Ψ_s_ decreased much faster for both Cabernet Volos and Fleurtai; therefore, samples from 22 and 44 days after beginning of water stress were selected.

### 2.2 RNA extraction and sequencing

Total RNA was extracted from about 50 mg of the powdered sample using a protocol implemented to obtain grapevine RNA of quality fit for high-throughput sequencing (Buesa et al., 2022; Dermastia et al., 2021). The CTAB (cetyltrimethylammonium bromide) buffer protocol was adapted from Carra et al. (2007) and combined with a Direct-zol™ RNA Miniprep Plus kit (Zymo Research).

Briefly, CTAB buffer (100 mM Tris-HCl, pH = 8.0, 2 M NaCl, 25 mM EDTA, 2.0% (w/v) CTAB, 2.5% (w/v) PVP40, 2.0% (v/v) β-mercaptoethanol) was warmed to 65 °C and added to the frozen powdered material. An equal volume of chloroform-isoamyl alcohol (24:1, choloroform: isoamyl alcohol) was added, thereafter the sample was vortexed and centrifuged for 10 min at 10.000 × g at 4 °C. The upper aqueous phase was collected and transferred to a new tube, to which 1.5 volume of pure ethanol was added to precipitate RNA at 4 °C for 30 min. The precipitate was then purified with Zymo-Spin Columns according to the manufacturer’s instructions, subjected to DNase digestion to remove DNA (DNase I Set, Zymo Research), and further purified with the RNA Clean & Concentrator kit (Zymo Research). RNA quality and quantity were evaluated using 2100 Bioanalyzer and RNA 6000 Nano Kit (Agilent Technologies). Libraries for RNA-Seq and Illumina HiSeq 4000 sequencing services were provided by Novogene (Hong Kong, China).

The150 bp paired-end reads obtained were trimmed to remove low-quality bases (Phred < 20), clipped to remove remaining adapter sequences and mapped to the 12X.2 version of the PN40024 grapevine reference genome (https://urgi.versailles.inra.fr/files/Vini/Vitis%2012X.2%20annotations/) using CLC Genomics Workbench 12.0 (Qiagen), with the following parameters: mismatch cost 2, insertion or deletion cost 3, length fraction 1.0, similarity fraction 0.95, and maximum number of hits for a read 1. The reads were annotated with the VCost.v3 annotation (Canaguier et al., 2017) and exported as raw counts.

Analysis of the exported raw counts was performed in R v4.0.2 (R Core Team, 2018), using the limma package v3.45.8 (Ritchie et al., 2015). Genes with low expression were removed by keeping only the genes with more than 50 counts in at least four samples. Data normalization to library sizes was done with Trimmed mean of M-values (TMM) normalization from the edgeR package v3.28.1 (Robinson et al., 2010). Normalized and log-transformed total count values were used to perform a principal component analysis (PCA) and PCA plots visualized with the ggbiplot package v0.55 (Wickham, 2016).

### 2.3 Preparation of leaf extracts and metabolite analysis

One hundred mg of leaf powder were incubated for 10 min at 25 °C in a 1 ml pre-chilled extraction mixture exactly described in (Hochberg et al., 2013). The mix was then centrifuged at full speed for 10 min (Eppendorf centrifuge 5424 R, DE), the supernatant recovered and added with 300 µl of chloroform and 300 µl of water, vortexed and then centrifuged for 5 min. 100 µl of upper phase of the supernatant was collected and dried in a speed-vacuum (Hetovac VR-1).

All the samples were then stored at -80 °C until analysis. Before analysis, all the samples were dried again for 30 min using Concentrator Plus (Eppendorf concentrator plus, DE) and derivatized as described by Hochberg et al. (2015). Residues were redissolved and derivatized for 120 min at 37 °C (in 40 μL of 20-mg/mL methoxyamine hydrochloride in pyridine) followed by a 30-min treatment with 70 μL N-methyl-N-(trimethylsilyl) trifluoroacetamide at 37 °C. Eight microliters of a retention time standard mixture (0.029% v/v n-dodecane, n-pentadecane, n-nonadecane, n-docosane, n-octacosane, n-dotracontane, and n-hexatriacontane dissolved in pyridine) were added before trimethylsilylation. The sample set also included an Arabidopsis thaliana quality control reference from a bulked extraction of Columbia-0 plants and a mixture of authentic metabolite standards (0.05 mg/ml).

Sample volumes of 1 μl were then injected into the GC column. The GC/MS system consisted of an AS 3000 autosampler, a TRACE GC ULTRA gas chromatograph, and a DSQII quadrupole mass spectrometer (Thermo-Fisher ltd) as described in Hochberg et al. (2015). The mass spectrometer was tuned according to the manufacturer’s recommendations using tris-(perfluorobutyl)-amine (CF43). GC was performed on a 30-m VF-5 ms column with 0.25 mm i.d., film thickness of 0.25 μm, and + 10 m EZ-Guard (Agilent). A 1-μl sample was injected into an injection port liner (Split liner with Wool, Restek, USA). The use of a programmable temperature vaporizer (PTV) enabled control of the injection temperature gradient from 60 °C to 300 °C at a rate of 14.5 °C/s, the transfer line was 300 °C, and the ion source was adjusted to 250 °C. Helium set at a constant flow rate of 1 ml/min was the carrier gas. The temperature program comprised 1 min of isothermal heating at 70 °C, a 1-°C/min oven temperature ramp to 76 °C, then a 6-°C/min oven temperature ramp to 350 °C, and finally, 5 min of heating at 350 °C. Mass spectra were recorded at eight scans per second with a mass-to-charge ratio 70 to 700 scanning range. Acquired spectra were then searched for using the National Institute of Standards and Technology (NIST, Gaithersburg, USA) algorithm incorporated in the Xcalibur^®^ data system (version 2.0.7) against standard RI libraries (http://gmd.mpimp-golm.mpg.de/) and in-house standard libraries. The data of each chromatograph was normalized by the internal standard ribitol.

### 2.4 Data integration

**Figure 1:**
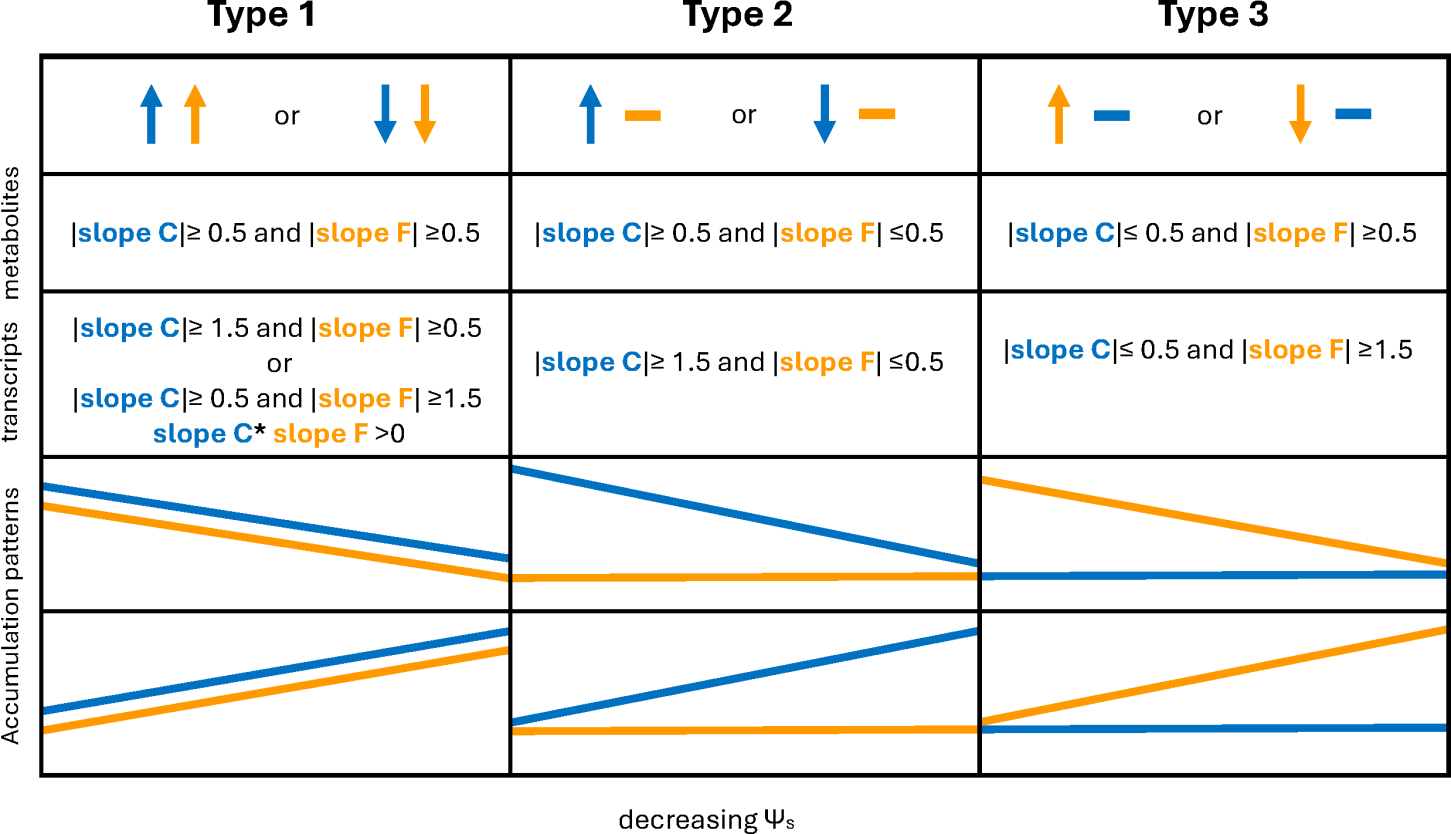
Representation of the three categories grouping similarly regulated genes and metabolites. Slope of the regression line describes the relation between gene expression or metabolite accumulation and Ψ_s_. Type 1 represents genes and metabolites that respond similarly in both genotypes. Type 2 represents genes and metabolites that respond only in Cabernet Volos. Type 3 represents genes and metabolites that respond only in Fleurtai. The metabolite and transcript panels show the formulae used to categorize the transcripts or metabolites in the three types. The accumulation pattern panels represent the general pattern of slopes, characteristic of each type. C, Cabernet Volos; F, Fleurtai.

The relation of transcripts and metabolites to Ψ_s_ was analyzed in R v4.0.2. The counts were first filtered to exclude genes that did not exceed 50 reads in at least four samples and normalized to sample library size. Principal component analysis (PCA) was performed using the PCA function from the FactoMineR package (v1.0.3) (Lê et al., 2008). For further analysis, we developed the FaDSeqSes (Factorial design of RNA-Seq analyses) script available at https://github.com/NIB-SI/FaDSeqSes. For each sample, the Ψ_s_ was estimated by linear interpolation of measured values. Gene expression was analyzed by linear model (lmFit) function from the R *limma* package (Ritchie et al., 2015) using a multifactor linear model, incorporating genotype and Ψ_s_. The rate of change in gene expression for each individual gene was represented by the slope of the model. Genes with absolute slope values greater than 1.5 were considered to have a strong change in expression and genes with absolute slope values less than 0.5 were considered not to have changed substantially. Genes with absolute slope values between 0.5 and 1.5 were considered to have a moderate change in expression. Based on gene expression and metabolite accumulation profiles, three categories of changed expression or abundance were identified and translated into threshold values for regression slopes in the two genotypes. The first category, named Type 1, contains genes that were either up-regulated by low Ψ_s_ or down-regulated by low Ψ_s_ in both genotypes. In this category, the regression line of at least one of the genotypes showed absolute value of the slope larger than 1.5 and for the other genotype larger than 0.5. Type 2 included genes that were up- or down-regulated in genotype Cabernet Volos but not in Fleurtai, and Type 3 included genes that were up- or down-regulated in genotype Fleurtai, but not in Cabernet Volos. In Type 2 and Type 3 categories, absolute values of the slopes of regression lines for the first genotype were above 1.5, while for the other genotype they were lower than 0.5. A schematic summary of the characteristics of each category is shown in **Figure 1**.

Metabolites were also classified in the same three categories, Type 1, 2, and 3, while the thresholds for the categories were set differently due to the lower slopes of regression lines. In Type 1 the regression lines of both genotypes possessed absolute value of the slope larger than 0.5; and in categories Type 2 and 3, absolute values of the slopes for the first genotype were above 0.5, while for the other genotype lower than 0.5. To compare the abundance of metabolites between the two genotypes, a difference between the metabolite amount for Fleurtai and Cabernet Volos was calculated for the low stress (Ψ_s_ = 0) and moderate stress (Ψ_s_ = -1) conditions.

Pearson’s correlation was used to assess the relationship between transcript and metabolite abundance and declining Ψ_s_. To focus on the most biologically relevant comparisons, the correlation was made on a subset of genes in the same GoMapMan functional categories (BINs) as the metabolites.

## 3 Results

### 3.1 Changes in physiological parameters under water stress

**Figure 2:**
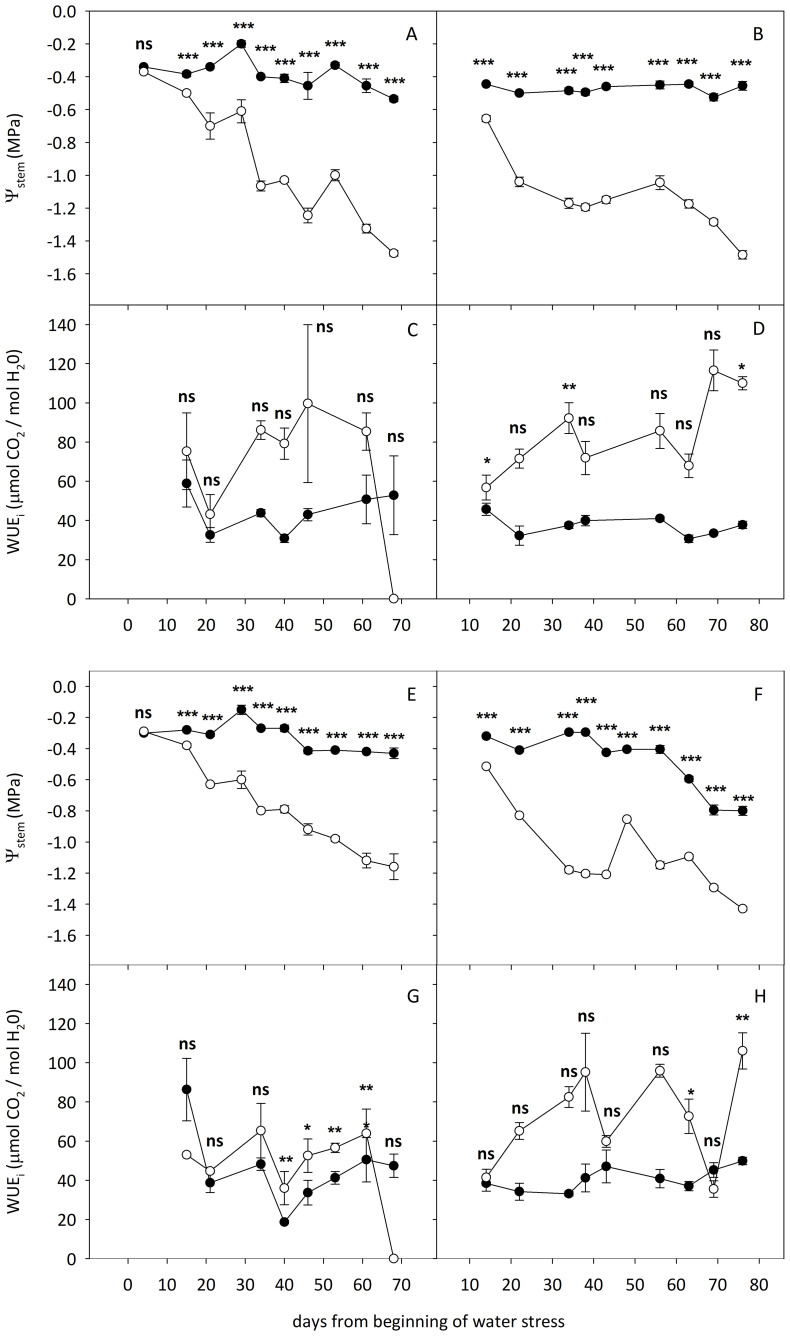
Midday stem water potential (Ψ_s_) and water use efficiency (WUE_i_) measurements. Pannels **(A–D)** Measurements for Cabernet Volos. Pannels **(E–H)** Measurements for Fleurtai. Stem water potential **(A, B, E, F)** and WUE_i_ **(C, D, G, H)** of WW (black symbols) and WS (empty symbols) vines in 2018 **(A, C, E, G)** and 2019 **(B, D, F, H)**. X-axis values are expressed relative to T0, which represents the onset of water stress. At each date, t-test was applied to determine the significance of differences between means (n = 4; ns, not significant; *, p < 0.05; **, p < 0.01; ***, p<0.001). WW, well-watered; WS, water stress.

The vines’ physiological parameters mirrored the different trends in temperatures in the two seasons. In both Cabernet Volos and Fleurtai well-watered (WW) plants, the stem water potential (Ψ_s_) was maintained constantly above the critical value of -0.6 MPa in both seasons (**Figures 2A, B, E, F**). In water-stressed (WS) plants, Ψ_s_ dropped constantly reaching a critical value of -1.4 MPa in Cabernet Volos and -1.2 MPa in Fleurtai only at the end of the season 2018; while in 2019 the decrease in Ψ_s_ was much faster because of the heatwaves in June and July (**Supplementary Figure 1**). In 2019, Fleurtai needed additional irrigation at the end of July since Ψ_s_ fell below -1.2 MPa that was set as the lower limit for this variety.

A_n_ in WS vines was more reduced for Cabernet Volos, related to the more severe water stress imposed for this variety. Contrarily, g_S_ showed similar trends of differences between WW and WS in both varieties (**Supplementary Figures 2**, **3**). Lastly, WUE_i_ was improved in case of WS vines for both varieties, but Cabernet Volos showed slightly greater differences during the central part of water constrain period as compared to Fluertai in both years, proving to perform better in conditions of water stress (**Figures 2C, D, G, H**).

### 3.2 Water stress-induced metabolic shift and transcriptional reprogramming in leaves

Thirty-two out of 55 identified metabolites changed their accumulation in water stress conditions in grapevine leaves. These metabolites are related to several different biological processes including photosynthesis, major and minor CHO metabolism, TCA cycle, cell wall composition, lipid, nitrogen, amino acid, nucleotide and secondary metabolism, and redox reactions (**Supplementary Table 1**).

Out of the 42,413 genes annotated to the grapevine genome (PN40024, 12X), in mature leaves the expression of 16,005 and 15,562 genes was detected in 2018 and 2019, respectively (**Supplementary Tables 2A, B**); expression of 15,242 of these genes was identified in both seasons, while expression of 763 and 320 genes was specific in 2018 and 2019, respectively.

**Figure 3:**
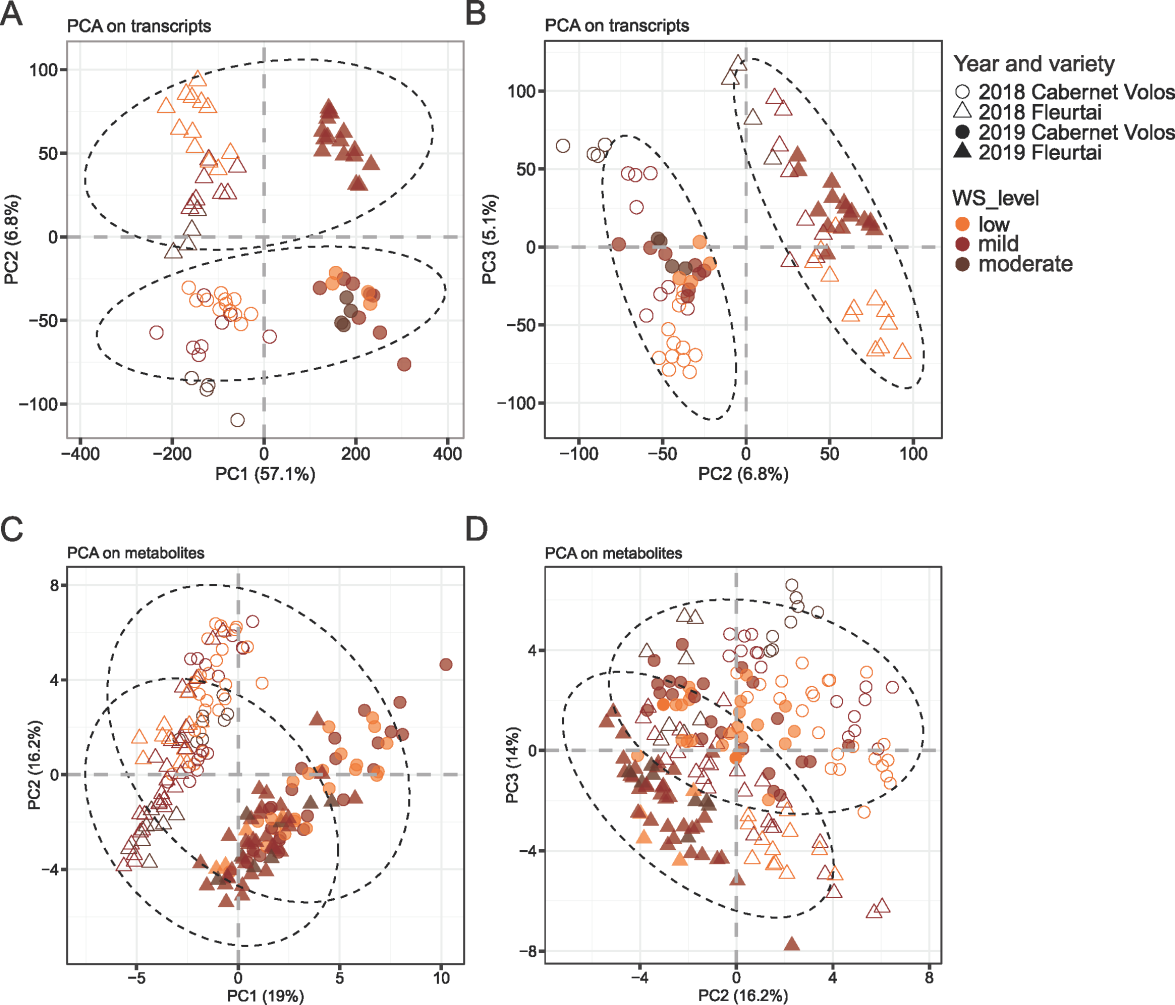
Principal Component Analysis (PCA) of transcripts and metabolites. PCA scatter plots show sample distribution based on transcript reads **(A, B)** and metabolite concentrations **(C, D)** in leaves of Cabernet Volos (circles) and Fleurtai (triangles) collected in 2018 (empty symbols) and 2019 (filled symbols). Samples are colored according to stress severity: low stress – orange, mild stress – red, moderate stress – brown. Categories of stress levels according to Ψ_s_: low stress- samples with Ψ_s_ > -0.5 MPa for Cabernet Volos and Ψ_s_ > -0.4 MPa for Fleurtai; mild stress - samples with -0.5 ≤ Ψ_s_ < 1.3 MPa for Cabernet Volos and -0.4 MPa ≤ Ψ_s_ < 1.15 MPa for Fleurtai; moderate stress - samples with Ψ_s_ ≤ -1.3 MPa for Cabernet Volos and Ψ_s_ ≤ -1.15 MPa for Fleurtai.

Principal Component Analysis (PCA) showed that both transcripts and metabolites were most prominently separated by the year of experiment (PC1), followed by genotype and water stress level (PC2) measured as Ψ_s_ (**Figure 3**). The separation according to water stress level was clearer for transcripts than for metabolites.

Aiming to identify the genes exhibiting expression consistent with metabolite accumulation, we analyzed transcriptomic and metabolomics data using a multifactor linear model with interactions of genotype and Ψ_s_. Based on gene expression and metabolite accumulation profiles in relation to decreasing Ψ_s_, three categories were identified (Type 1, Type 2, Type 3), as detailed in **Figure 1**.

Among 19 metabolites of Type 1 (similar accumulation patterns in response to water deficit in both cultivars), sugars and amino acids prevailed. The greatest influence of water deficit on both cultivars surfaced in the accumulation of amino acids phenylalanine, leucine, proline, isoleucine, valine and gamma-aminobutyric acid (GABA), and sugars raffinose, galactinol, and melibiose (**Table 1**). Shikimate, a precursor of polyphenol synthesis, exhibited the most prominent decrease with the reduction of Ψ_s_.

**Table 1:** List of identified metabolites.

Type	Metabolite	Slope C	Slope F	Diff LS	Diff MS
Type 1	Glyceric acid	-0.63	-0.96	-0.83	-0.35
Type 1	Fructose-6-phosphate	-0.69	-0.77	-0.06	0.06
Type 1	Sucrose	-0.66	-0.89	0.21	0.56
Type 1	Galactinol	0.94	1.10	0.86	0.62
Type 1	Raffinose	1.28	1.74	1.43	0.74
Type 1	Threitol	0.55	0.53	0.07	0.10
Type 1	Erythronic acid	0.66	0.88	0.30	-0.03
Type 1	Citric acid	0.62	0.79	-0.06	-0.32
Type 1	Melibiose	1.20	1.36	0.39	0.15
Type 1	Proline	0.86	1.64	0.47	-0.71
Type 1	Valine	0.73	1.08	0.57	0.04
Type 1	Leucine	1.17	1.68	0.49	-0.28
Type 1	Isoleucine	0.60	1.43	0.70	-0.54
Type 1	Shikimic acid	-1.59	-1.54	0.91	0.83
Type 1	Phenylalanine	1.16	1.89	0.82	-0.28
Type 1	Mannose-6-phosphate	-0.52	-0.66	-0.12	0.09
Type 1	GABA	0.80	2.36	1.46	-0.87
Type 1	Threonolactone	-0.98	-0.86	-0.54	-0.73
Type 1	Tartaric acid	-0.50	-0.76	-0.70	-0.30
Type 2	Aspartic acid	0.62	-0.33	-1.08	0.35
Type 2	Hydroquinone	-0.50	-0.45	1.03	0.95
Type 2	Threonic acid	-0.84	-0.24	0.17	-0.74
Type 2	Putrescine	1.00	0.04	-1.10	0.34
Type 2	Maleic acid	0.55	0.42	0.24	0.44
Type 2	Phosphoric acid	-0.99	-0.09	1.39	0.05
Type 3	Glucose	-0.25	-0.56	0.22	0.68
Type 3	Alanine	0.44	0.58	0.42	0.22
Type 3	Succinic acid	0.02	0.52	0.95	0.20
Type 3	Rhamnose	0.41	0.83	0.99	0.38
Type 3	Glutamic acid	0.46	0.54	-0.01	-0.13
Type 3	3-caffeoylquinic acid	-0.23	1.13	7.52	5.48
Type 3	Ascorbic acid	-0.41	-0.61	0.83	1.12
/	Glycine	0.35	0.19	0.10	0.34
/	Serine	-0.09	-0.38	0.08	0.52
/	Fructose	0.15	0.46	1.33	0.87
/	Myo-Inostol	-0.11	-0.23	0.43	0.62
/	Galactose	-0.02	-0.24	0.30	0.62
/	Xylose	-0.45	-0.22	0.28	-0.06
/	Malic acid	-0.21	-0.34	0.33	0.52
/	Fumaric acid	0.33	0.22	0.28	0.45
/	Arabinose	-0.25	-0.17	0.39	0.27
/	Ribonic acid	-0.02	0.14	0.04	-0.20
/	Ribose	-0.26	-0.10	0.18	-0.07
/	Ethanolamine	0.11	0.35	0.57	0.22
/	Threonine	0.47	-0.04	-0.35	0.42
/	Trans-Caffeic acid	-0.24	-0.07	0.38	0.12
/	Quinic acid	-0.19	-0.38	2.13	2.42
/	Gallic acid	-0.23	-0.03	0.28	-0.02
/	Catechin	-0.46	-0.23	3.93	3.58
/	Pyroglutamic acid	0.48	0.02	-0.12	0.56
/	Uracil	-0.18	-0.28	0.14	0.29
/	Gluconic acid	-0.05	0.16	-0.27	-0.59
/	Lyxonic acid	-0.32	-0.44	-0.08	0.10
/	Malonic acid	-0.19	-0.35	-0.16	0.09
/	Glucopyranose[-H20]	0.05	0.03	0.63	0.67

Slope C - slope of the regression line of the relation between metabolite amount and Ψ_s_ in Cabernet Volos; Slope F - slope of the regression line of the relation between metabolite amount and Ψ_s_ in Fleurtai; Diff LS - difference between regression lines of the relation between metabolite amount and Ψ_s_ in Cabernet Volos and Fleurtai at well-watered conditions (Ψ_s_ = 0); Diff MS - difference between regression lines of the relation between metabolite amount and Ψ_s_ in Cabernet Volos and Fleurtai at moderate water deficit conditions (Ψ_s_ = -1). Blue and red indicate decreased and increased metabolite accumulation, respectively. Yellow indicates the difference between the points of regression lines.

**Figure 4:**
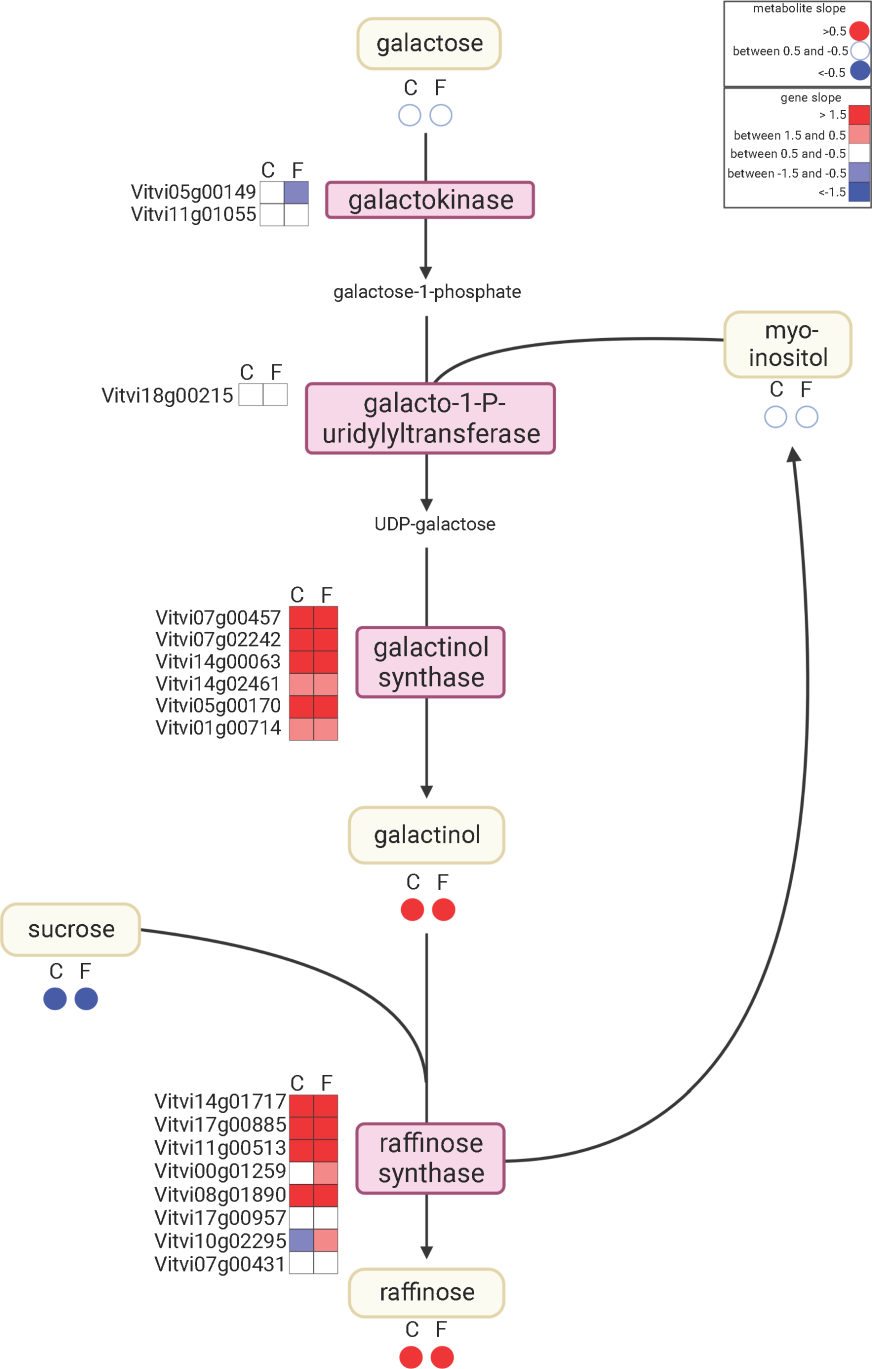
Raffinose synthesis pathway showing transcript and metabolite measurements. Slopes of the regression lines relating to metabolite abundance and Ψ_s_ (metabolite slope), and gene expression and Ψ_s_ (gene slope) are presented as color-coded scales for Cabernet Volos (C) and Fleurtai (F). Red represents increased abundance in relation to increasing Ψ_s_, blue represents decreased abundance in relation to increasing Ψ_s_. Metabolites are presented as circles, transcripts are presented as squares.

The 689 genes assigned to Type 1 belonged to 22 different GoMapMan gene sets based on their predicted function (**Supplementary Table 3**). Minor carbohydrates metabolism and amino acid metabolism were among the most represented functional groups. Moreover, a significant positive correlation was found between the accumulation of raffinose and the expression of raffinose synthase genes (*Vitvi14g01717, Vitvi11g00513, Vitvi17g00885*) and between galactinol and various galactinol synthase genes (*Vitvi07g00457, Vitvi07g02242, Vitvi14g02461, Vitvi05g00170, Vitvi14g00063, Vitvi01g00714*) (**Figure 4**; **Supplementary Figure 4**).

We identified several upregulated Type 1 genes related to abscisic acid synthesis, including nine-cis-epoxycarotenoid dioxygenase and aldehyde oxidase (*Vitvi19g01356*, *Vitvi02g01288*, and *Vitvi18g02167*), as well as ABA-inducible genes (*Vitvi03g01727* and *Vitvi17g00085*). Conversely, genes downregulated by the decreasing Ψ_s_, were related to auxin, ethylene, and gibberellin signal transduction (*Vitvi14g01899*, *Vitvi09g00050*, *Vitvi01g02286*, *Vitvi16g00349*, *Vitvi07g02064*, *Vitvi16g00350*, and *Vitvi17g00601*) and gibberellin, cytokinin, and salicylic acid metabolism (*Vitvi16g00890*, *Vitvi19g02230*, *Vitvi14g03084*, *Vitvi18g01019*, and *Vitvi04g02118*) (**Supplementary Table 3**).

The change in Ψ_s_ also caused changes in the expression of genes involved in transcriptional regulation. Fifteen transcription factors were upregulated as Ψ_s_ decreased, including some genes in the MYB, NAC, and bZIP families, while twenty-eight genes were repressed by the decreasing Ψ_s_, including some genes in the WRKY, basic helix-loop-helix (bHLH), and MYB families. Several genes pertaining to protein synthesis and modification were also modulated, most being upregulated (**Supplementary Table 3**). We also found considerable downregulation in functional groups related to cell wall metabolism, primarily genes associated with cell wall degradation and modification (**Supplementary Table 3**).

We found upregulation of genes encoding biotic stress receptors, such as osmotins (*Vitvi02g01408* and *Vitvi02g01409*), and other abiotic response proteins such as small HSPs in both varieties. By contrast, several biotic stress associated genes showed downregulation in response to water deficit; the majority were PR proteins, *PR5* being the most represented (*Vitvi02g00391*, *Vitvi02g01404*, and *Vitvi17g00217*) (**Supplementary Table 3**).

Finally, Type 1 contained a large group of 48 kinase and receptor kinase genes, all of which, except four, were downregulated as Ψ_s_ decreased (**Supplementary Table 3**). Interestingly, the latter group was also most represented among the Type 2 which contains genes responsive to Ψ_s_ in Cabernet Volos and not in Fleurtai. Kinases and receptor kinases accounted for 27 out of 63 Type 2 genes (**Supplementary Table 4**).

Type 3 contained 41 genes that were significantly expressed in response to Ψ_s_ decrease in Fleurtai, but not in Cabernet Volos. Among those, 26 genes were upregulated and 15 downregulated due to decreased Ψ_s_ (**Supplementary Table 3**). Genes related to secondary metabolism represented the largest upregulated group: three terpene synthases genes (*Vitvi19g00391* and *Vitvi18g02446*, *Vitvi12g00574*) and two UDP-Glycosyltransferases genes (*Vitvi15g01075* and *Vitvi15g01643*). Additionally, a GCR2-like 1 encoding gene involved in ABA signaling (*Vitvi00g02022*) and a gene encoding a disease resistance protein (TIR-NBS-LRR class) (*Vitvi18g01877*) were upregulated. Transport-related genes were also represented in this category, including an ABC transporter, a cation efflux protein, and sulphate transporters (*Vitvi09g00303*, *Vitvi19g01715*, *Vitvi07g01779*).

**Figure 5:**
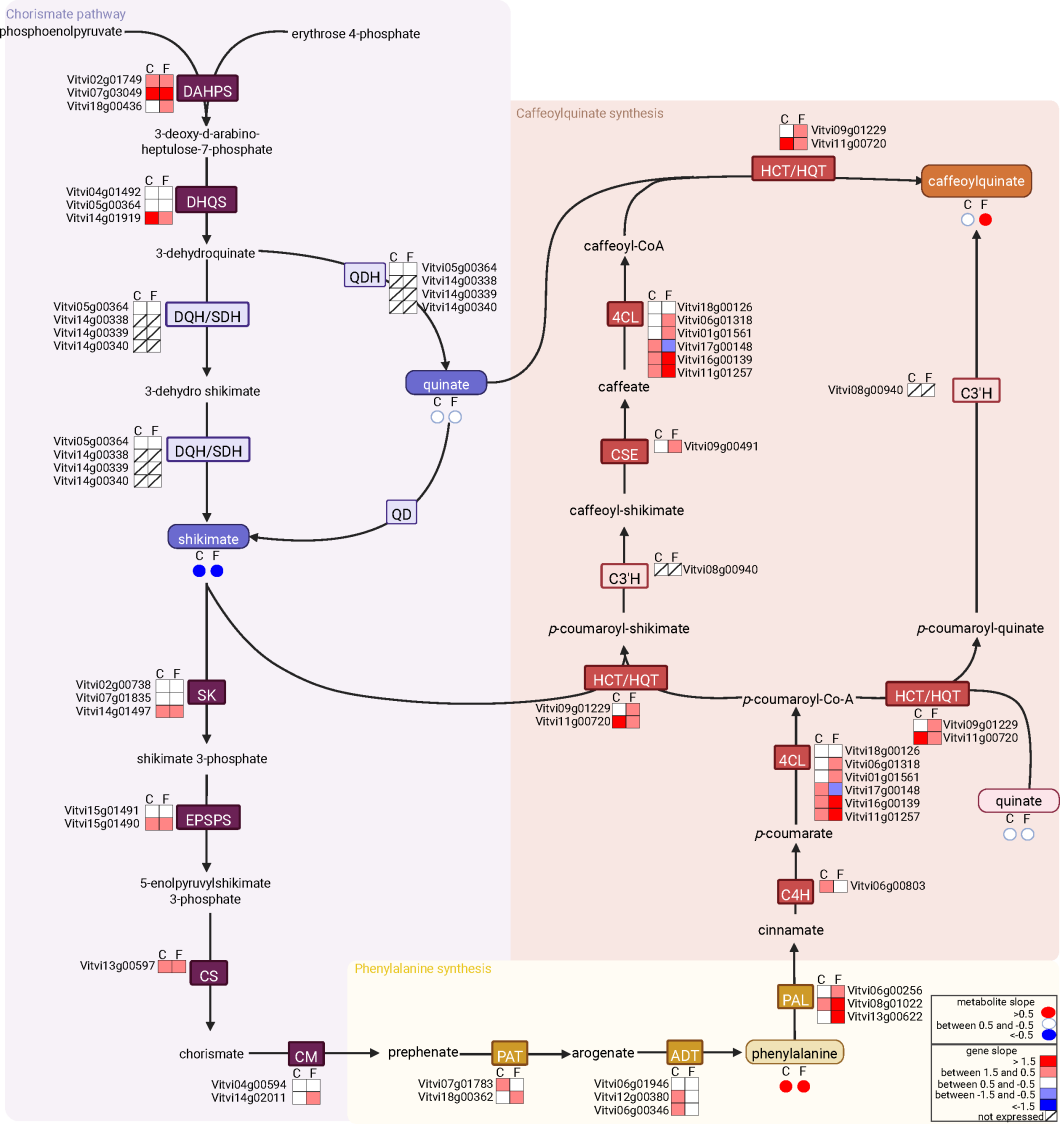
Caffeoylquinate synthesis pathway showing transcript and metabolite measurements. Slopes of the regression lines relating to metabolite abundance and Ψ_s_ (metabolite slope), and gene expression and Ψ_s_ (gene slope) are presented as color-coded scales for Cabernet Volos (C) and Fleurtai (F). Red represents increased abundance in relation to increasing Ψ_s_, blue represents decreased abundance in relation to increasing Ψ_s_. Metabolites are presented as circles, transcripts are presented as squares.

The content of shikimate, a precursor of phenylalanine and polyphenols, decreased during the experimental period, while several genes involved in the biosynthesis of shikimate, for instance 3-deoxy-D-arabino-heptulosonic acid 7-phosphate synthase gene (*DAHPS*, *Vitvi07g03049, Vitvi02g01749)*, and 3-dehydroquinate synthase gene (*DHQS*, *Vitvi14g01919*) showed an increase in their transcript level in both genotypes (**Table 1**, **Figure 5**). These results suggested a rapid catabolism steering the shikimate pool toward the biosynthesis of phenylalanine and downstream metabolites. Consistently, several genes involved in the chorismate pathway and phenylalanine synthesis were upregulated with the decreasing Ψ_s_, e.g., shikimate kinase (*Vitvi14g01497*), 5-enolpyruvylshikimate 3-phosphate synthase (*EPSPS*) (*Vitvi15g01490*), and chorismate synthase (*Vitvi13g00597*) (**Figure 5**). Quiniate abundance remained unchanged regardless Ψ_s_ in both Fleurtai and Cabernet Volos, while caffeoylquinate showed a considerable increase in Fleurtai with the drop of Ψ_s_. This, together with the negative correlation found between *DHQS* and quinate suggests an increase in caffeoylquinate biosynthesis (**Figure 5**).

Other amino acids also exhibited correlations with genes related to their biosynthesis. For instance, we found positive correlation between proline and delta 1-pyrroline-5-carboxylate synthase 2 (*Vitvi13g00355*) and between GABA and a glutamate decarboxylase (*Vitvi04g00870*) in both genotypes (**Supplementary Figure 4**).

## 4 Discussion

Grapevine adopts several strategies to cope with water shortage, ranging from a limitation of growth to stomatal closure and finally leaf shedding (Lovisolo and Schubert, 1998; Gerzon et al., 2015; Degu et al., 2019; Falchi et al., 2019). Nonetheless, in the field large differences in water stress tolerance occur among grapevine genotypes. Moreover, the impact of water scarcity on their growth is expected to increase due to climate change, affecting both viticultural practices, e.g. cultivars and locations suited for viticulture (Rossdeutsch et al., 2016; van Leeuwen et al., 2024). At the same time, *Plasmopara viticola* remains a persistent threat to vineyards even in very dry regions, as it can survive long periods of and multiply extremely rapidly as favorable conditions arise (Cohen et al., 2020; Viret and Gindro, 2025).

Reduced water availability affects plant physiology by both morphological and transcriptional modifications (Dal Santo et al., 2016; Gambetta et al., 2020; Hochberg et al., 2023). Deloire et al. (2004) indicated that stem water potential (Ψ_s_) values lower than -1.4 MPa are considered critical for grape production, as they could cause physiological damage and atypical maturation of grapes; for white or sensitive varieties, the limit is raised to -1.2 MPa to maintain the aromatic profile. For these reasons, our experimental design consisted of non-irrigated plots with water being added only if the above-mentioned limits were reached. As expected, water stress caused a constant reduction of Ψ_s_ and g_S_ with similar trends in both cultivars.

Limited knowledge exists about water stress tolerance of newly developed grapevine hybrids resistant to biotic stresses, deriving from only a few recent studies. Braidotti et al. (2024) investigated how two fungi-resistant varieties (Sauvignon Kretos and Merlot Kanthus) responded to water deficits and found that under moderate stress, each cultivar exhibited notable differences in stomatal conductance that were more cultivar, than stress-dependent. The same was also true for the leaf metabolite profile. By measuring ABA and the expression of genes involved in its perception, they indicated that ABA was water deficit-responsive, but each genotype displayed different levels of ABA sensitivity. In contrast to the findings in this study, they observed and increase in salicylic and jasmonic acid. de Almeida et al. (2024) also demonstrated that water deficit responses are highly cultivar specific. They measured indicators of primary metabolism and thiol precursors in berries of Syrah and six different disease-resistant varieties. They demonstrated that water deficit negatively affected primary metabolism and thiol precursor accumulation in a similar manner for most genotypes. The exception was 3159-B, which preferentially accumulated thiol precursors under water deficit. In this study, we set out to broaden the knowledge of how disease-resistant varieties respond to water deficits by capturing a bigger picture of the transcriptome and metabolome state of stressed plants.

We utilized both transcriptomic and metabolomics approaches to unveil molecular pathways of water deficit response in two disease-resistant grapevine varieties, Fleurtai and Cabernet Volos which were not previously investigated for this trait. The factorial design of analyses by FaDSeqSes script, implemented here, took advantage of the availability of gene expression, metabolite accumulation, and Ψ_s_ data from the same vines. Namely, the environmental factors in the experimental years differed substantially, leading us to shift our focus from comparing WS and WW grapevine data. Instead, Ψ_s_ values were directly related to gene expression and metabolite accumulation levels. We performed linear regression for each gene or metabolite to assess how Ψ_s_ influences its expression or accumulation levels in each of the two genotypes.

Factorial analysis of gene and metabolite behavior in Cabernet Volos and Fleurtai resulted in three different behavior types. In Type 1, where transcripts, metabolites and accumulation pattern behavior was the same between both genotypes, we categorized 19 metabolites and 689 genes as important in grapevine response to water stress. It is not surprising that this group encompassed several upregulated sugars and amino acids; transcripts involved in carbohydrate metabolism, cell wall synthesis, modification and degradation, lipid metabolism, amino acid metabolism, secondary metabolism and hormone metabolism, as it is well known that reallocating resources is a part of the plants’ strategy to adapt to water stress (Dace et al., 2023). Furthermore, highest Pearson correlation values between transcripts and metabolites was found for raffinose family oligosaccharides, a group of sugars involved in the response to drought and desiccation in different plant organs (Angelovici et al., 2010; ElSayed et al., 2014; Sengupta et al., 2015). Several studies carried out in grapevine revealed that changes occur in these metabolic pathways in response to drought stress (Chaves and Oliveira, 2004; Dal Santo et al., 2016; Catacchio et al., 2019; Cochetel et al., 2020).

Notably, both Fleurtai and Cabernet Volos showed modulation of genes related to osmotic stress response (galactionol and raffinose synthesis, GABA and proline metabolism, osmotins), to abscisic acid hormone synthesis or ABA-inducible genes (i.e. *HVA22*), and the transport of ABA and amino acids, confirming the common tissue-specific adaption mechanisms acting in the primary response to drought stress (Catacchio et al., 2019). Metabolomic analysis further revealed an accumulation of osmoprotectants (i.e. sugars and amino acids) in the drought stress response of both genotypes, in agreement with previous reports for grapevine and other plant species (Nahar et al., 2016; Haider et al., 2017; Sharma et al., 2019). Compatible solutes such as sugar alcohols (e.g., galactinol) and amino acids function in osmotic adjustment, preserving plant cell turgor under stress conditions. Proline has a well-known function as a compatible solute, and increasing its accumulation through overexpression of its main biosynthesis gene (*P5CR*) has shown to increase drought resistance in both transgenic Arabidopsis and grapevine (Chen et al., 2021; Zhang et al., 2023). Several studies have reported the increased importance of both proline and ABA in grapevine response to the combination of heat and drought stress (Lehr et al., 2022; Tan et al., 2023). Moreover, these compatible solutes, especially galactinol and proline, can also act as free radical (ROS) scavengers (Takahashi et al., 2020). Similarly, GABA, a non-protein amino acid, accumulates in plants under stress (Kinnersley and Turano, 2000; Jia et al., 2013) to mitigate drought-depressed cell elongation and wax biosynthesis (Li et al., 2021).

It is very likely that conserved metabolic pathways related to central metabolism, including sugars and their derivatives, amino and organic acids, involved in major C-N and energy metabolism, measured by the GC-MS protocol used, respond similarly between species and even more so between varieties (Hochberg et al., 2013). Urano et al. (2009) have shown metabolite accumulation in Arabidpopsis leaves exposed to water stress that resemble the results found in the current study, including the accumulation of raffinose family oligosaccharides, GABA and proline. Urano proposed an ABA dependent transcriptional regulation of metabolic accumulation in response to drought, which is consistent with the many upregulated ABA related genes found in our data. This universal metabolic response of central metabolism and the accumulation of metabolites under stress is at least in part a result of reduced growth, i.e. an accumulation led by the decreased consumption of C-N backbones (Obata and Fernie, 2012).

Members of bZIP, MYB and NAC transcription factor families were found upregulated in both Fleurtai and Cabernet Volos in this study, confirming their role in the regulation of plant drought responses (Hirayama and Shinozaki, 2010). Interestingly, among them we found *VvNAC26* (*Vitvi01g01038*), a transcription factor that has been cloned from *V. amurensis* (a cold- and drought-hardy *Vitis* species) and was demonstrated to improve drought tolerance in transgenic *Arabidopsis* (Fang et al., 2016). The same gene was up-regulated in both root and shoot in response to water deficit in *V. riparia* (Khadka et al., 2019).

Downregulated transcripts we found in common in both genotypes (Type 1) were mostly related to cell wall metabolism, degradation and modification (**Supplementary Table 3**). Several studies are consistent with this finding, reporting that cellulose biosynthesis can be altered in response to water deficit, as shown by the decline in synthesis and a clear shift in the cell wall composition in grape leaves under drought conditions (Sweet et al., 1990; Le Gall et al., 2015).

In addition, several biotic stress associated genes, mostly PR proteins (*PR5* and PR5-like receptor kinases being the most represented) showed downregulation in response to water deficit. Haider et al. (2017) found similar results reporting that, conversely to heat shock proteins, most of the PR showed down-regulation in grapevine leaves under drought stress, suggesting that water deficit has a negative effect on the defense response. Increasing evidence suggests that leucine rich repeat kinases could regulate abiotic and biotic stress responses and have a major role in integrating environmental and plant hormone signaling (Osakabe et al., 2013), with variable regulation according to genotype and timing (Yan et al., 2023). In our study, we observed some upregulation of these kinases, however, downregulation predominated, which indicates a tradeoff between abiotic and biotic stress responses.

Downregulation of biotic defense related genes was also a marked feature in the Type 2 gene category, which contains genes that were responsive in Cabernet Volos, but not in Fleurtai (**Supplementary Table 6**). Out of the 63 genes found in Type 2, 24 were annotated as LRR and PR5-like receptor kinases. Most of the other genes in this category were downregulated as well, with one particularly interesting upregulated gene being a GDSL-motif lipase (*Vitvi09g00038*). The same gene has been identified by transcriptomic network analyses of leaf dehydration responses in Cabernet Sauvignon, Riparia Gloire, and Ramsey genotypes (Hopper et al., 2016). Even if the exact role of the GDSL lipases has not been clarified in grapevine, several studies ascribe them to plant stress adaption and leaf water retention, as they are involved in cutin biosynthesis (Li et al., 2017; Tang et al., 2020).

As for Fleurtai-specific transcriptomic and metabolomic changes in response to water stress (41 Type 3 genes) occurred mainly in hormone signal transduction and secondary metabolism (**Supplementary Table 3** and **5**). Cabernet Volos had a high, but constant level of caffeoylquinate regardless of treatment. At the same time, Fleurtai increased the accumulation of caffeoylquinate in response to water deficit, but never reached the levels recorded in Cabernet Volos. Caffeoylquinate is a plant phenolic compound that is believed to be an intermediate in lignin biosynthesis. Due to its antioxidative properties, it is an important protective molecule for plants in many different environmental stresses, including low temperature, drought, and high light (Chen et al., 2025). Hochberg et al., 2013 showed that caffeoylquinate was specifically associated to drought in the Syrah metabolic network. In conjunction, an accumulation of suberin in the vascular bundles was reported as well. Similarly in this study, the phenylpropanoid pathway was strongly induced, hinting that vascular remodeling may be a large part of adaptation in Fleurtai and Cabernet Volos genotypes as well. Moreover, in both genotypes strong upregulation of *CYP98A3* (Vitvi06g00605), involved in precursor synthesis for lignin and related compounds (Alber et al., 2019) was observed. The accumulation of flavonoid compounds in response water deficits is a commonly noted phenomenon, although it seems that it depends strongly on the stress duration and plant developmental phase. For example, Tan et al. (2023) found that Cabernet Sauvignon exposed to a combination of heat and drought stress showed very similar transcript-level responses observed in our experiments, however, they reported a marked decrease in the expression of genes related to the phenylpropanoid synthesis pathway. This could be related to the duration of stress, as well as the use of self-rooted one year old cuttings in their experiments.

We found several terpene synthase encoding genes that were uniquely upregulated in Fleurtai, not surprising given the role of terpenes in mitigating drought stress in plants (Yadav et al., 2021). This is consistent with the idea that in white cultivars, terpenes and carotenoids compensate take over the protective function anthocyanins play in red varieties during stress response.

A GCR2-like 1 encoding gene (*Vitvi00g02022*), was also upregulated in Fleurtai. This gene is involved in ABA signaling pathway, central to drought stress perception and has a recognized role in drought tolerance (Almagro et al., 2014; Priya et al., 2019; Arisha et al., 2020; Yang et al., 2021). Although belonging to the Type 1 category, the *CYP707A4* (*Vitvi18g00792*), crucial for ABA catabolism (Saito et al., 2004), exhibited a much higher degree of induction in Fleurtai. Gaiotti et al. (2023) reported that Moscato Giallo controlled its stomata more tightly in response to drought and had higher ABA sensitivity compared to the more anisohydric varieties.

## 5 Conclusions

Our study identified both common and genotype-specific pathways for drought avoidance in the two cultivars. In response to water deficit, both would activate the accumulation sugars and amino acids, cell wall related processes, and secondary and hormone metabolism. Cabernet Volos exhibited a prominent shut down of biotic defense and signaling related genes, while Fleurtai activated genes involved in secondary metabolism and transport. This was also confirmed by the different water potential values reached by the two cultivars under the same water regime. Given our findings we hypothesize that Cabernet Volos accumulates osmoprotectant molecules for cellular osmotic adjustment and mitigation of damage caused by ROS at a higher level, both before and during water deficit, which enables a larger stem water potential drop than in Fleurtai. Fleurtai however has a greater propensity to synthesize these compounds at a higher rate as the stress arises. The overview provided by the transcriptomic analysis indicates that the response to water stress is cultivar-specific, and also very intricate, involving a huge number of coordinated transcriptional changes. The results from this study unquestionably confirm the findings from (Hopper et al., 2016) hinting that ABA signaling pathway has a crucial role in leaf dehydration response with several differences among grapevine genotypes. Future research is needed to elucidate the regulation of these gene networks and to possibly provide a functional validation of genes or metabolic pathways identified, with a view to assist future breeding programs in the context of different biotic and abiotic stress combinations that are expected to arise due to climate change.

## Data Availability

The datasets presented in this study can be found in online repositories. The names of the repository/repositories and accession number(s) can be found below: https://www.ebi.ac.uk/ena, ERS6293633, ERS6293634, ERS6293635, ERS6293636, ERS6293637, ERS6293638, ERS6293639, ERS6293640, ERS6293641, ERS6293642, ERS6293643, ERS6293644, ERS6293645, ERS6293646, ERS6293647, ERS6293648, ERS6293649, ERS6293650, ERS6293651, ERS6293652, ERS6293653, ERS6293654, ERS6293655, ERS6293656, ERS6293657, ERS6293658, ERS6293659, ERS6293660, ERS6293661, ERS6293662, ERS6293663, ERS6293664, ERS6293665, ERS6293666, ERS6293667, ERS6293668, ERS6293669, ERS6293670, ERS6293671, ERS6293672, ERS6293673, ERS6293674, ERS6293675, ERS6293676, ERS6293677, ERS6293678, ERS6293679, ERS6293680, ERS6293681, ERS6293682, ERS6293683, ERS6293684, ERS6293685, ERS6293686, ERS6293687, ERS6293688, ERS6293689, ERS6293690, ERS6293691, ERS6293692, ERS6293693, ERS6293694, ERS6293695, ERS6293696, ERS6293697, ERS6293698, ERS6293699, ERS6293700, ERS6293701, ERS6293702, ERS6293703, ERS6293704, ERS6293705, ERS6293706, ERS6293707, ERS6293708, ERS6293709, ERS6293710, ERS6293711, ERS6293712.
