# Supplementary material for: Combined omics data reveal multilevel response to water deficit in grapevine disease-resistant varieties
Source: Front Plant Sci. 2026 Jul 8;17:1818883. doi: 10.3389/fpls.2026.1818883 (PMC13389845; doi:10.3389/fpls.2026.1818883)
Supplement: Supplementary file 1 [file DataSheet1.zip › supplements/Suplementary figures 1, 2 and 3.pdf]

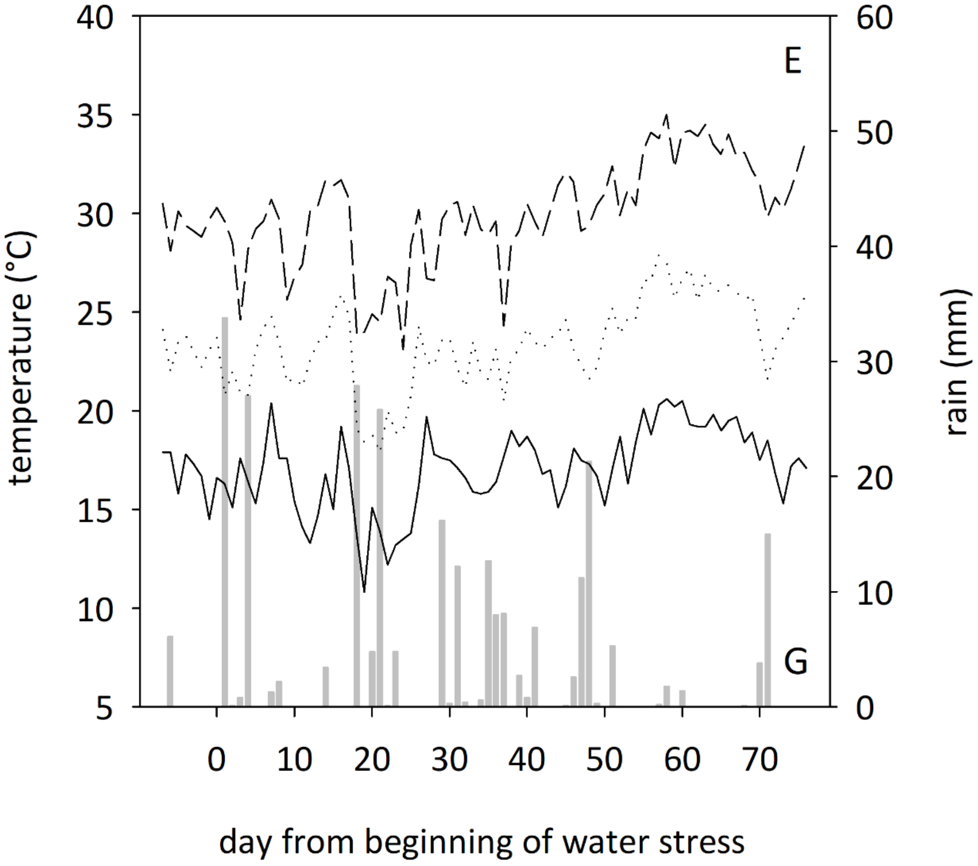

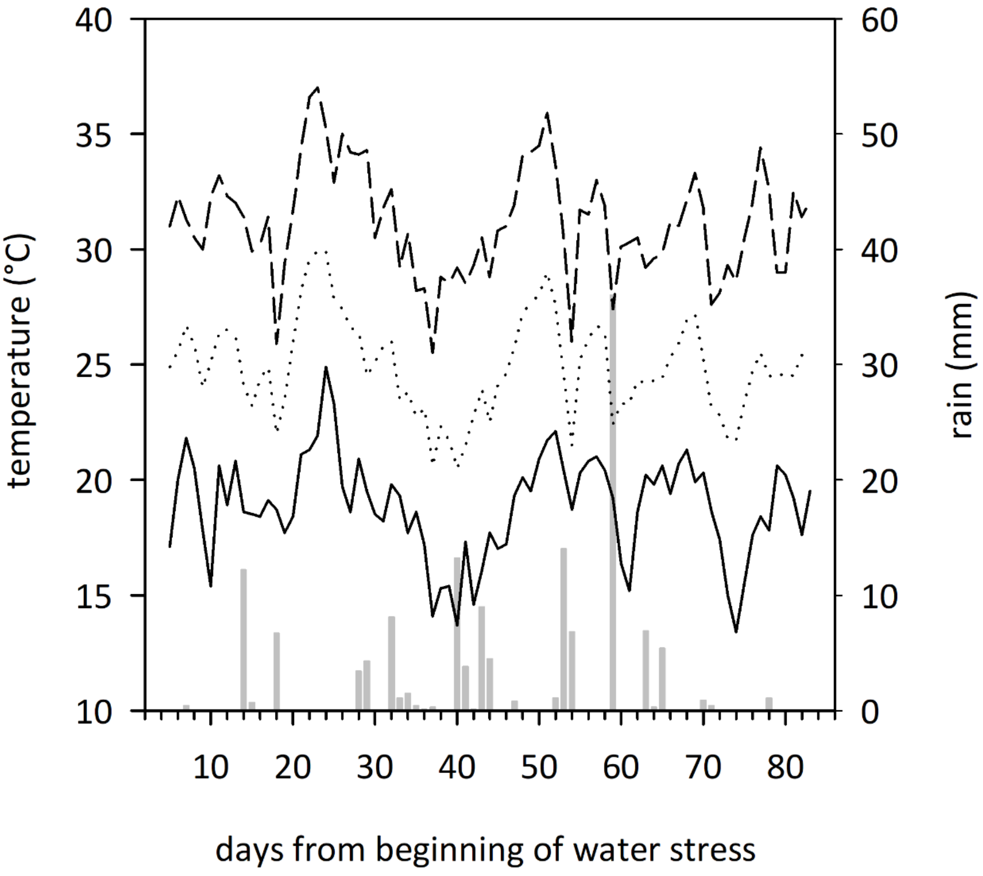

Supplemental Figure 1: Temperature and rainfall measurements for 2018 (above) and 2019 (below). Data recorded by the weather station of Udine S. Osvaldo (ARPA FVG–OSMER, http://www.meteo.fvg.it/).

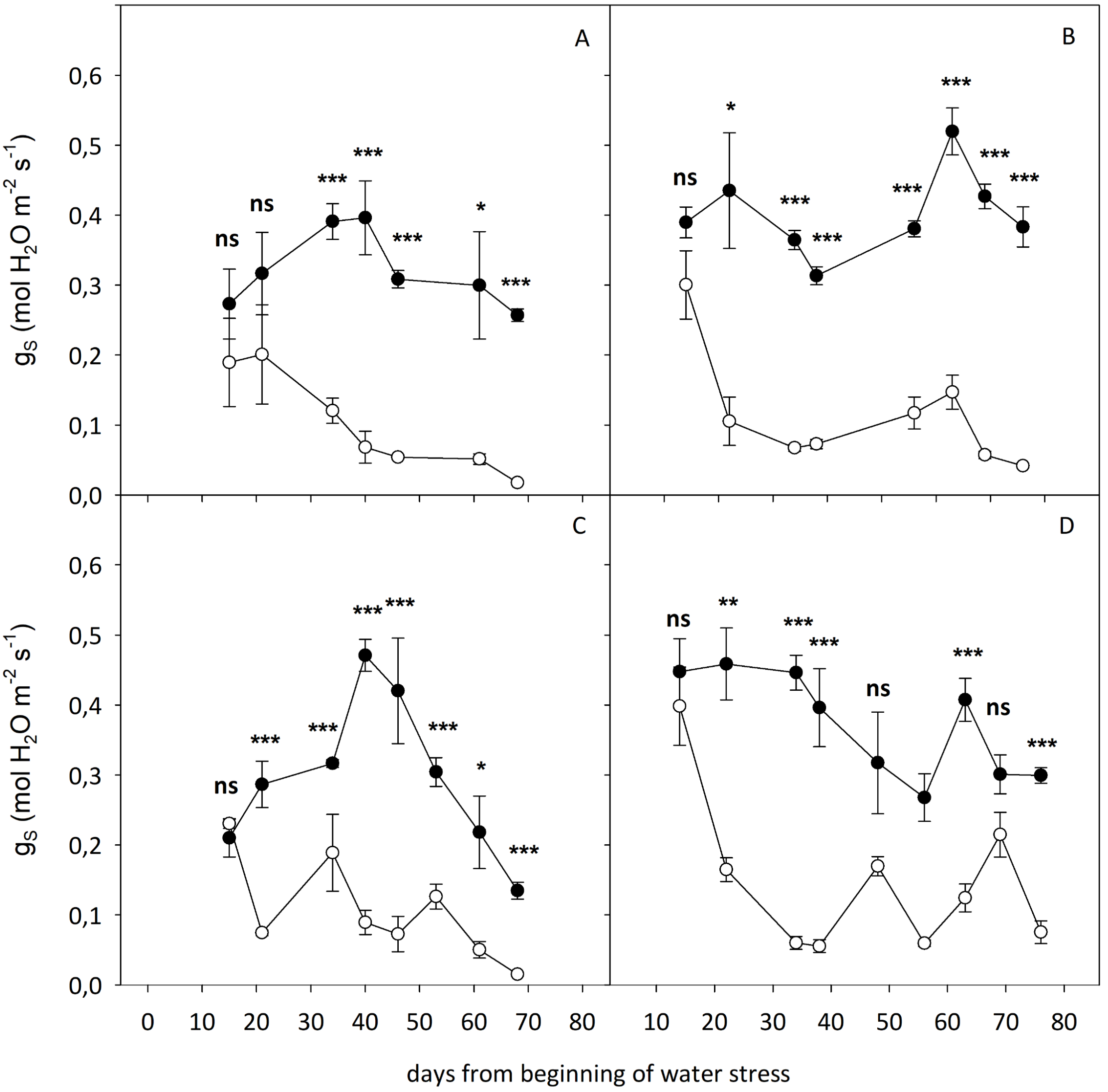

Supplemental Figure 2: Stomatal conductance of WW (black symbols) and WS (empty symbols) vines of cultivar Cabernet Volos (A and B) and Fleurtai (C and D) in 2018 (A and C) and 2019 (B and D). X-axis values are days expressed relative to T0, which represents the onset of water stress. At each date, t-test was applied to determine the significance of differences between means (n = 4; ns, not significant; *, $p < 0.05$; **, $p < 0.01$; ***, $p<0.001$). WW, well-watered; WS, water stress.

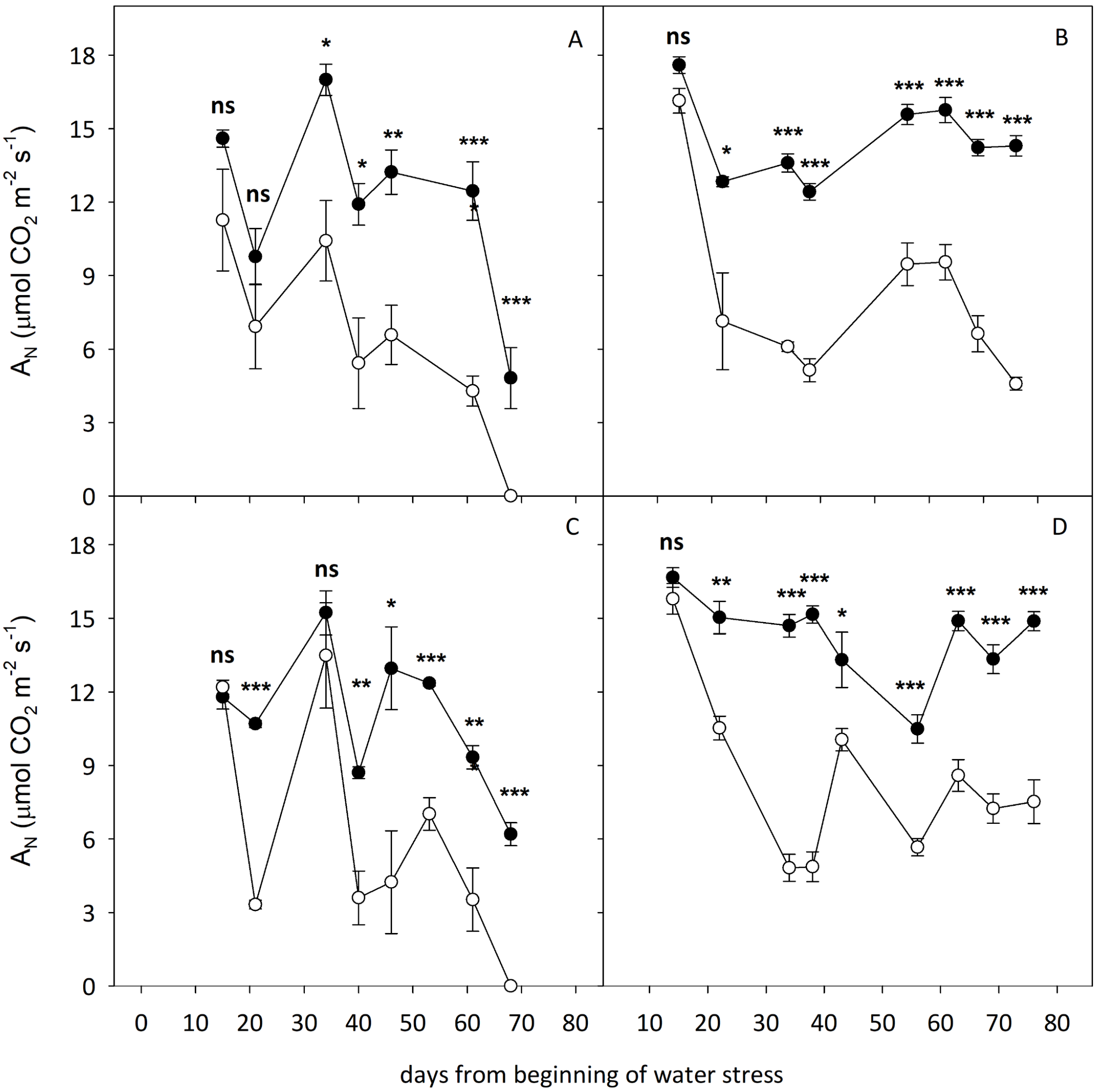

Supplemental Figure 3: CO_2 assimilation rate of WW (black symbols) and WS (empty symbols) vines of cultivar Cabernet Volos (A and B) and Fleurtai (C and D) in 2018 (A and C) and 2019 (B and D). X-axis values are days expressed relative to T0, which represents the onset of water stress. At each date, t-test was applied to determine the significance of differences between means (n = 4; ns, not significant; *, $p < 0.05$; **, $p < 0.01$; ***, $p<0.001$). WW, well-watered; WS, water stress.
